# Quality of sleep and health-related quality of life among health care professionals treating patients with coronavirus disease-19

**DOI:** 10.1177/0020764020942800

**Published:** 2020-07-16

**Authors:** Jelena Stojanov, Marina Malobabic, Goran Stanojevic, Milos Stevic, Vuk Milosevic, Aleksandar Stojanov

**Affiliations:** 1 Special Hospital for Psychiatric Disorders ‘Gornja Toponica’, Nis, Serbia; 2 Clinic of Neurology, Clinical Center Nis, Nis, Serbia; 3 Medical Faculty, University of Nis, Nis, Serbia

**Keywords:** COVID-19, pandemic, quality of sleep, quality of life

## Abstract

**Background:**

Health care professionals exposed to coronavirus disease 2019 (COVID-19) are facing high levels of stress.

**Aim:**

The aim was to evaluate the quality of sleep (QoS) and health-related quality of life (HRQoL), among health care professionals treating patients with COVID-19, as well as quantifying the magnitude of symptoms of depression and levels of anxiety.

**Methods:**

We included 201 health care professionals in a cross-sectional, web-based study by applying 7-item Generalized Anxiety Disorder (GAD-7) Scale, Zung Self-rating Depression Scale, 36-item Health Survey of the Medical Outcomes Study Short Form (SF36), Pittsburgh Sleep Quality Index (PSQI) and additional survey constructed for the purpose of the study.

**Results:**

Health care workers who treated COVID-19 patients were more afraid of becoming infected or of transmitting the infection to a family member with a significantly low self-assessment of their mental status. Poor QoS and HRQoL correlated with high health anxiety and severe depressive symptoms and several demographic characteristics. Multiple linear regression analysis showed that higher scores on GAD-7 (beta = .71, *p* < .01) and lower scores on mental health (MH) subscale on SF36 questionnaire (beta = –.69; *p* < .01) were independent predictors of the higher PSQI score (adjusted *R*^2^ = .61, *p* < .01 for overall model). Higher scores on GAD-7 (beta = .68, *p* < .01) and worse self-perceived mental status (beta = .25; *p* < .05) were independent predictors of the lower SF36 scores (adjusted *R*^2^ = .73, *p* < .01 for overall model).

**Conclusion:**

The major MH burden of health care professionals treating infected patients during the COVID-19 pandemic indicates that they need psychological support.

## Introduction

In March 2020, a novel infective disease caused by a coronavirus disease 2019 (COVID-19) was first reported in Serbia, after becoming a global health emergency. Series of restrictive measures were introduced, and one of them was the overall organization of work in the form of ‘Work From Home’, which could not be applied to health care professionals.

Health care professionals reported having the moral obligation to treat patients and save lives (Greenberg et al., 2020). They were at the time of writing the article on the front line, in the designated COVID-19 and other hospitals, and directly involved in the fight against the pandemic, despite elevated personal risk. Being in an unprecedented situation included taking a risk by making difficult decisions and work under extreme pressures. The way they cope with pandemic determines not only their physical health, but also their mental health (MH). Fear, anxiety, and depression are emotions that arise in response to stress, but they are primarily cognitive constructs. Previous studies report that health care professionals feared of being infected felt stigmatized, experienced high levels of anxiety and symptoms of depression, and had sleep problems (Lai et al., 2020). Also, poor quality of sleep (QoS) is associated with high levels of anxiety and symptoms of depression in the general population (Yeen & Ning, 2020). COVID-19 pandemic was also associated with impaired health-related quality of life (HRQoL) among local residents (Y. Zhang & Ma, 2020). HRQoL and QoS are associated with cognitive assessments of well-being, as seen from the individual’s perspective. There is not enough data on the HRQoL and QoS in health care professionals during the COVID-19 pandemic. Impaired HRQoL and QoS can disrupt their efficiency in providing medical services and may lead to a reconsideration of the chosen call. It is important not to lose the sense of what they are doing and for them to have the experience that they are not alone in the fight. An insight into the consequences of their cognitive processing would make it easier to provide adequate psychological support.

The aim of the current study was to evaluate HRQoL and QoS among health care professionals treating patients with COVID-19, as well as quantifying the magnitude of symptoms of depression and levels of anxiety and by analyzing potential risk factors associated with these symptoms.

## Methods

### Study design, approval and procedures

To prevent the spread of infection, we performed a cross-sectional, web-based study after 20 days of the establishment of Temporary Hospitals to combat the COVID-19 pandemic, to collect data. Verbal informed consent was provided by all survey participants before their enrollment. The survey was anonymous, and confidentiality of information was assured. The study began by uploading the electronic version of the survey to the participants’ emails with a brief explanation of the survey and its purpose. Clicking the link automatically opened the survey. They were allowed to terminate the survey at any time. This web-based survey was completely voluntary and non-commercial.

The study was conducted in accordance with the approval from the clinical research ethics committee of the local review board and all applicable guidelines, including the Basics of Good Clinical Practice, the Declaration of Helsinki and the Law on Health Care of the Republic of Serbia.

### Participants

Health care professionals who worked in Clinical Center Nis, Serbia, were asked to participate. During the COVID-19 pandemic in this Institution, four wards were designed to work with COVID-19 positive patients. Health care professionals assigned to work in a Temporary Hospital worked in a ward with mild to moderate COVID-19 positive patients with the highest level of protection and with full equipment in shifts to a maximum of 4 hours and a maximum of eight shifts per week. By the time the article was written, more than 1,000 patients were treated in Temporary Hospitals in Nis, Serbia, a larger number were in wards, where health care professionals in our sample were deployed. Health care professionals working on other departments during the COVID-19 pandemic in Clinical Center Nis and not dealing with COVID-19 patients were also asked to participate. They were sex- and age-matched, and were used as a control group. Excluding factors were the existence of sleep–wake disorders or any other psychiatric disorders prior to COVID-19, which included any changes in mental status due to the need for psychiatric care before the pandemic, as well as the use of therapy for the psychiatric disorders. We used random sampling to reduce risk of bias. Approximately, 300 health care professionals worked with COVID-19 positive patients during the pandemic, and to 181, the online survey was sent. Total number of 118 was included in the study; 49 were excluded due to exclusionary factors and others refused to participate or did not answer on our call. In other departments, there were approximately 1,200 health care workers, a survey form was sent to those who had an email address available to us at that time (138 of them). Total number of 83 health care workers was included in the study; others were excluded due to exclusion factors or did not answer on our call.

### Survey

The survey consists of the following parts: part incorporating demographic characteristics, part of COVID-19-related data constructed for the purposes of this study and part consisting of standardized and validated questionnaires in Serbia.

Basic demographic data included occupation (doctor or nurse), gender (male or female), age (years), marital status (single, married or cohabitant), living area (urban or rural), an education level (secondary school, university degree, postdoctoral degree), presence of other chronic diseases that demand chronic use of certain drug therapies, number of children, partner’s employment status, presence of psychiatric disorders in the family and so on. COVID-19-related data included questions about attitudes toward current needs, overall satisfaction with support provided and importance of psychosocial support, whether they or their family members or close friends were diagnosed with COVID-19, questions about the attitude and respect toward restrictive measures and the attitude of others toward them given their exposure to the virus, including being stigmatized.

Self-reported participants’ MH status was measured by 7-item Generalized Anxiety Disorder (GAD-7) Scale, Zung Self-rating Depression Scale (SDS), 36-item Health Survey of the Medical Outcomes Study Short Form (SF36) and Pittsburgh Sleep Quality Index (PSQI).

The GAD-7 scale (range 0–21) is used to assess the severity of symptoms of anxiety. GAD-7 results are interpreted as follows: normal (0–4), mild (5–9), moderate (10–14) and severe (15–21) anxiety. Using the threshold score of ⩾10 (high anxiety), the GAD-7 has a sensitivity and specificity of several anxiety and stress-related disorders, but further evaluation is recommended (Spitzer et al., 2006). The GAD-7 has been previously used in Serbian populations and found to have good reliability (Lapcevic et al., 2017).

The SDS is a 20-item self-report questionnaire used as a screening tool for depression, and the total score indicated the existence and intensity of existing depressive symptoms (Zung, 1965). The total score range is from 25 to 100. Normal range was classified with score 25–49, mild depression with score 50–59, moderate with score 60–69 and severe depression with score ⩾70. The SDS is standardized and validated and has been previously used in Serbian populations (Milic et al., 2019).

The SF36 Health Survey is a 36-item self-report survey that assesses eight domains of physical health and MH, ranging from 0 to 100, where the highest score indicates the optimal HRQoL and the lowest score indicates the poorest HRQoL. The eight domains are physical functioning (PF), role limitations because of physical health problems (role-physical (RP)), bodily pain (BP), general health (GH) perceptions, vitality (VT), social functioning (SF), role limitations because of emotional problems (role-emotional (RE)) and general MH (Ware & Sherbourne, 1992). The first four domains (PF, RP, BP and GH) constitute the physical health and the other four domains (VT, SF, RE and MH) constitute the MH (Milic et al., 2020). The SF36 is widely used in health research and is validated and tested for reliability by several studies in Serbia (Ilic et al., 2020; Stojanov et al., 2019; Ware & Sherbourne, 1992).

PSQI QoS questionnaire consists of 19 self-rated questions. These 19 questions are grouped into seven groups, each scored from 0 to 3. Obtained global PSQI score is from 0 to 21, where a higher score indicates the lower QoS. Components of the questionnaire measure QoS, duration and latency of sleep, common efficacy of sleep and functionality during the day. It assesses a 1-month interval and provides data useful in both clinical and scientific works. In addition to assessing the QoS, it provides a clinically useful evaluation of a variety of factors that might affect the QoS. It could be used as a tool for measuring the interaction of sleep disturbances and levels of anxiety, the intensity of depressive symptomatology, as well as the relationship between sleep quality and other demographic characteristics (Buysse et al., 1989). It is a widely used QoS survey, which is translated and standardized in Serbia (Mollayeva et al., 2016; Popevic et al., 2018).

### Statistical analysis

All data were statistically processed by the IBM SPSS statistical software (version 21) for the Windows operative system. First, descriptive analyses were conducted to describe the demographic characteristics and COVID-19-related data in health care workers who treated COVID-19 patients. The Mann–Whitney test was used to compare continuous variables between two groups, and the Kruskal–Wallis test was used to compare more than two groups. Correlations were assessed using Pearson’s correlation coefficients or Spearman’s correlation coefficients. Values of *p* < .05 were considered statistically significant (two-sided tests). Numerical data are presented as medians and interquartile range (IQR) for nonparametric data and as mean ± standard deviation (*SD*) for parametric data. The multivariate logistic regression models were performed to explore potential influence factors for worse QoS and quality of life (QoL).

## Results

### Demographic characteristics and COVID-19-related data of health care professionals who treated COVID-19 patients

The demographic characteristics of our participants are presented in Table 1. The self-assessment of the current mental state indicated that in the group of medical workers who worked with COVID-19 positive patients, 18.5% rated their mental status as excellent, 24.5% as very good, 42.3% as good and 14.7% as bad. Compared with pre-pandemic mental status, worsening was reported by 64.3% of subjects, and a total of 61.5% of participants rated the pandemic as having a negative impact on their MH. The biggest reported fear was the fear of infection (65.3%), the fear that someone close to them could get infected (83.3%), and the fear that they might infect a family member (59.5%). Other reasons were present in less than 15% (fear of death caused by the COVID- 19, fear that someone close to them might die, fear that their previously present health problems could worsen, etc.). A total of 67% of participants stated that they were bothered by restrictive measures (police lockdown, quarantine and social isolation), and 96.4% also stated that they respected the legally prescribed measures. In 9.7% of participants, family members were infected, while 69.7% had acquaintances that were infected with COVID-19. Only 7.5% of participants stated that they need the professional support of a psychologist/psychiatrist during a pandemic, while 12.7% stated that they have problems that they want to solve on their own. Others assessed that they do not need professional psychological support.

**Table 1.** Socio-demographic characteristics of health care workers who treated (Group I) and did not treat (Group II) coronavirus disease 2019 patients.

	Group I (*N* = 118)	Group II (*N* = 83)
Age, years (*M* ± *SD*)	39.1 ± 7.3	42.5 ± 9.7
Female gender (%)	65.6	66.3
Area of living – urban (%)	78.6	75.4
Education (%)		
Secondary studies	32.4	36.5
University degree	56.3	54.8
Postdoctoral studies	11.3	8.7
Partner status – married or cohabitant (%)	63.7	61.6
Occupation – nurse (%)	59.8	62.4
Number of children (%)		
Zero	43.7	40.6
One or two	50.2	52.2
Three or more	6.1	7.2
Presence of other chronic disease (%)	17.4	22.7
Partner occupation status – employed (%)	73.4	69.5
Family member lost job due to pandemic (%)	15.3	12.7
Psychiatric disease in family (%)	14.6	16.3

*SD*: standard deviation.

### Difference between the group of health care workers who worked with COVID-19 patients and those who worked on other departments during COVID-19 pandemic

Scores on obtained questionnaires are presented in Table 2. We noticed a statistically significant difference between two groups on scores for QoS and HRQoL (and also some subscores on SF36 questionnaires). Worst scores on SF36 subscales were obtained on the scale for MH and SF in both groups. Levels of anxiety and depression were also high. GAD-7 score ⩾10 was noticed in 31.8% of patients in a group of health care workers who treated COVID-19 and 16.4% of subjects in other groups of health care workers. Also, SDS score ⩾60 was noticed in 17.6% of participants in COVID-19 health care workers and 13.2% in other groups of health care workers. It was observed that health workers dealing with COVID-19 were significantly more afraid of becoming infected or of transmitting the infection to a family member (*p* < .01). Also, the self-assessment of their mental status was significantly worse than in the other group of health workers (*p* < .05). There was no significant difference compared with other examined parameters.

**Table 2.** PSQI, SF36, GAD-7 and SDS scores of health care workers who treated (Group I) and did not treat (Group II) coronavirus disease 2019 patients.

Variable	Group I (*N* = 118)	Group II (*N* = 83)
PSQI global score**	8.3 ± 4.5	5.2 ± 3.7
SF36 total score*	80.06 ± 24.69	86.14 ± 25.13
SF36 physical functioning	87.23 ± 25.42	88.23 ± 24.52
SF36 role-physical	82.13 ± 24.64	84.14 ± 23.53
SF36 bodily pain	91.14 ± 25.31	92.55 ± 24.84
SF36 general health	79.25 ± 29.14	81.53 ± 25.42
SF36 vitality*	77.31 ± 24.53	81.31 ± 25.63
SF36 social functioning	64.68 ± 24.63	67.25 ± 24.61
SF36 role-emotional	70.15 ± 24.61	72.31 ± 24.63
SF36 mental health*	59.48 ± 24.35	64.21 ± 24.64
GAD-7**	13.26 ± 5.32	8.25 ± 5.61
SDS*	53.14 ± 11.41	49.39 ± 10.61

*SD*: standard deviation; PSQI: Pittsburgh Sleep Quality Index; SF36: 36-item Health Survey of the Medical Outcomes Study Short Form; GAD-7: 7-item Generalized Anxiety Disorder Scale; SDS: Zung Self-Rating Depression Scale.

* *p* < .05; **p < .01.

### Influence of socio-demographic factors, anxiety and depression on scores obtained on questioners for QoS and QoL

A statistically significant correlation between variables and scores on SF36 and PSQI is presented in Table 3. Multiple linear regression analysis included all factors that correlated with higher PSQI scores showed that higher scores on GAD-7 (beta = .71, *p* < .01) and lower scores on MH subscale on SF36 questionnaire (beta = –0.69; *p* < .01) were independent predictors of the higher PSQI score (adjusted *R*^2^ = .61, *p* < .01 for overall model). Regarding lower total score on SF36, higher scores on GAD-7 (beta = .68, *p* < .01) and worse self-perceived mental status (beta = .25; *p* < .05) were independent predictors of the lower SF36 scores (adjusted *R*^2^ = .73, *p* < .01 for overall model).

**Table 3.** Correlation between variables and scores obtained on PSQI and SF36 among health care workers who treated COVID-19 patients (*N* = 118).

	SF36	PSQI
GAD-7	−0.62**	0.58**
SDS	−0.59**	0.51**
SF36 total	–	0.69**
SF36 mental health	–	0.71**
PSQI	−0.55**	–
Female	0.33*	0.31*
Education	0.13	0.25*
Married with children	0.24*	0.16

PSQI: Pittsburgh Sleep Quality Index; SF36: 36-item Health Survey of the Medical Outcomes Study Short Form; GAD-7: 7-item Generalized Anxiety Disorder Scale; SDS: Zung Self-Rating Depression Scale.

* *p* < .05; **p < .01.

## Discussion

This cross-sectional survey enrolled 201 health care professionals and revealed various reasons for concern about the MH of physicians and nurses working in Temporary Hospitals and treating infested patients during the COVID-19 pandemic. Our concern was also based on previous research data about the most prevalent long-term psychological condition among those at the front line in previous epidemics like SARS and MERS, as well as during COVID-19 pandemic in China (Lai et al., 2020; Lee et al., 2018; Mak et al., 2009).

High-stress situations like pandemic are followed by psychological responses. Although almost 70% assessed the restrictive measures during the pandemic as restrictive, the vast majority adhered to them. Facts and concerns about the mode of transmission and spread of potentially fatal virus bring people into a state of heightened vulnerability. Health care workers, in particular, are not feeling comfortable whenever they are not able to recognize, explain, predict and control disease processes, which have an impact on their MH (Kang et al., 2020). In more than 30% of health care professionals who treated patients with COVID-19, we registered high health anxiety and in nearly 20% of scores on SDS were ⩾60. Reported fears are in agreement with the registered fears in the studies of the same group of respondents (Lai et al., 2020). Health care workers dealing with COVID-19 were significantly more afraid of becoming infected or of transmitting the infection to a family member.

Health care professionals who treated COVID-19 patients had poorer QoS which correlated with high health anxiety and severe depressive symptoms, female gender, being a nurse as well as poor HRQoL. Higher health anxiety and lower mental HRQoL were independent predictors of the poorer QoS. Previous findings support our results, that health care professionals were anxious because of close and frequent contact with positive patients and consequently had lower QoS when committing themselves to provide high-quality medical care for patients (Lai et al., 2020; Li et al., 2003; Shih et al., 2007; Wong et al., 2005). Female gender and being a nurse was also associated with lower QoS and high health anxiety during COVID-19 pandemic in China (Gao et al., 2020; Guo et al., 2016; Yeen & Ning, 2020).

High-exposure physicians and nurses had lower QoS, although their work engagement provided them with rest, which indicates that they were not able to rest adequately due to the chronic stress and psychological distress, as previously described (Lu et al., 2006; McAlonan et al., 2007). Some studies found a connection between low QoS and post-traumatic stress disorder (PTSD) symptoms, which can be expected as a consequence of a pandemic (Kobayashi et al., 2007).

Although health care workers around the world have received an updated guideline on how to handle the patients with COVID-19 and an adequate supply of medical protective items (including masks, glasses and suits), they worked with enormous pressure (W. R. Zhang et al., 2020). They worked with a reduced number of medical professionals, faced with possible shortages of critical care medical resources and personal protective equipment as well as clinician deaths. Also, the fact that they worked in conditions of increased risk in a state of emergency police lockdown during the pandemic with restrictive measures that included the reduction of social contacts has additionally affected their physical and emotional well-being. Our results show low HRQoL among health care professionals, especially among those who treated COVID-19 positive patients. Impaired HRQoL independently correlated with high anxiety, more severe depressive symptoms, poorer QoS and female gender, being married with children.

Besides the fact that less than 20% of health care professionals who treated COVID-19 patients assessed their current mental state as excellent, and more than 50% reported significant worsening with the negative impact of a pandemic, only 7.5% indicated the need for professional psychological support. Therefore, we believe that it is necessary to organize and to provide adequate psychological support to health care providers during the future life-threatening infectious disease outbreaks, as well as recovery programs aimed at empowering resilience and psychological well-being.

This study has several limitations. First, data and relevant analyses presented here were derived from a cross-sectional study and by self-report tools, limited to the COVID-19 outbreak and voluntary basis. Second, it was not possible to assess the participation rate, since it is unclear how many subjects received the link for the survey. Third, due to the sudden occurrence of the disaster, we were unable to assess an individual’s psychological conditions before the outbreak; we were just sticking to the exclusionary factors. Our main focus was on their subjective assessment of QoS and HRQoL in whom no sleep–wake disorder or any psychiatric disorder has been diagnosed until then. Fourth, larger sample size is needed to verify the results.

In summary, our study revealed a major MH burden of health care professionals treating infected patients in Serbia during the COVID-19 pandemic. High risk of displaying psychological issues followed by low QoS and HRQoL was observed among female health care professional nurses, who are married and have children, working in the front line at Temporary Hospitals dealing with positive patients. Better personalized MH protection, support and care should be provided to high-exposed health care professionals during future life-threatening infectious disease outbreaks from psychotherapists and psychiatrists.
